# Predictors of Neurological and Functional Recovery in Patients with Moderate to Severe Ischemic Stroke: The EPICA Study

**DOI:** 10.1155/2020/1419720

**Published:** 2020-05-01

**Authors:** Manuel Murie-Fernández, Mercedes Molleda Marzo

**Affiliations:** ^1^Integral Neurological Care Centre, Neurorehabilitation Unit, Plaza Europa, 9, 31119 Imárcoain, Navarra, Spain; ^2^Hospital Universitario Germans Trias i Pujol, Rehabilitation Unit, Carretera de Canyet, s/n, 08916 Badalona, Barcelona, Spain

## Abstract

**Background:**

Improving our knowledge about the impact of restorative therapies employed in the rehabilitation of a stroke patient may help guide practitioners in prescribing treatment regimen that may lead to better post-stroke recovery and quality of life.

**Aims:**

To evaluate the neurological and functional recovery for 3 months after an acute ischemic stroke occurred within previous 3 months. To determine predictors of recovery.

**Design:**

Prospective observational registry. *Population*. Patients having suffered acute moderate to severe ischemic stroke of moderate to severe intensity within the previous 3 months with National Institutes of Health Stroke Scale (NIHSS) score from 10 to 20, 24 hours after arrival at emergency room (ER).

**Methods:**

All prespecified variables (sociodemographic and clinical data, lifestyle recommendations, rehabilitation prescription, and neurological assessments) were assessed at three visits, i.e., baseline (D0), one month (M1), and three months (M3).

**Results:**

Out of 143 recruited patients, 131 could be analysed at study entry within 3 months after stroke onset with a mean acute NIHSS score of 14.05, decreased to 10.8 at study baseline. Study sample was aged 64.9 ± 13.8 years, with 49.2% of women. Neurorehabilitation treatment was applied to 9 of 10 patients from the acute phase and for three months with different intensities depending on the centre. A large proportion of patients recovered from severe dependency on activities of daily living (ADL) at D0 to a mild or moderate disability requiring some help at M3: mean NIHSS = 10.8 to 5.7; median modified Rankin Scale (mRS) = 4 to 3; Barthel index (BI) = 40 to 70; all *p* values < 0.001. Multivariate analyses integrating other regression variables showed a trend in favour of rehabilitation and revascularization therapies on recovery although did not reach statistical significance and that the positive predictors of recovery improvement were baseline BI score, time to treatment, and dietary supplement MLC901 (NurAiD™II). A larger percentage of patients with more severe stroke (NIHSS > 14) who received MLC901 showed above median improvements on mRS compared to control group at M1 (71.4% vs. 29.4%; *p* = 0.032) and M3 (85.7% vs. 50%; *p* = 0.058). Older subjects and women tend to have less improvement by M3.

**Conclusions:**

Our study in patients with moderate to severe stroke shows overall recovery on neurological and functional assessments during the 3 months of study observation. Apart from demonstrating traditional “non-modifiable” predictors of outcome after stroke, like age, sex, and stroke severity, we also detected association between the use of dietary supplement MLC901 and recovery.

## 1. Introduction

Every year, nearly 120,000 people suffer from stroke in Spain, half of whom are disabled or suffer life-threatening sequelae. Although mortality and disability have declined in the last 20 years in all age groups and in both sexes in Spain, their incidence is expected to increase by 27%, according to data from the Spanish Society of Neurology [1]. The data further highlight stroke as the second leading cause of death overall in Spain, the leading cause of death among women, and the leading cause of disability in adults. The estimated incidence rate of stroke is 141 (95% confidence interval (CI) 125 to 158) per 100,000 inhabitants [2, 3]. Incidence rate clearly increases with age, with a peak at or above 85 years of age, and in-hospital mortality is 14%. Currently, more than 330,000 Spanish people have limited functional capacity due to stroke. Stroke survivors are at high risk for recurrent stroke and cardiovascular disease due to the pronounced aging of the population and the increased prevalence of risk factors in an increasingly elderly population [4]. In an epidemiological study of 321 patients diagnosed with stroke admitted to the stroke unit of 16 hospitals throughout Spain and followed up for 1 year, the total average cost per year was estimated at €27,711 per patient. Direct healthcare costs amounted to an average of €8491 (of which 68.8% was due to inpatient costs) and nonhealthcare costs to €18,643 per patient per year (of which 89.5% was due to informal care) [5].

Apart from the establishment of prevention programs and hospital stroke units, treatment of stroke is still limited in many settings. Even when patients receive acute stroke therapies, they may not derive benefit from such therapies, and as a result, a significant proportion of patients suffer from chronic sequelae and persistent deficits that significantly impact their ability to carry out their daily activities and quality of life [6].

Restorative therapies that improve neural repair and recovery in the postacute phase of stroke may reduce the overall long-term burden of stroke [7]. Thus, during the post-stroke rehabilitation process, different interventions may be prescribed as part of the holistic approach to help patients recover, combining different types of cognitive and motor therapies, as well as changes in lifestyle and use of supplements and herbal medicines [8, 9]. Improving our knowledge about the impact of these therapies employed in the rehabilitation of a stroke patient may help guide practitioners in prescribing treatment regimen that may lead to better post-stroke recovery and quality of life.

This observational study aimed to assess the rates of neurological and functional recovery over a 3-month follow-up period in a cohort of patients who have experienced a moderate to severe first-ever acute ischemic stroke and to determine predictors of greater recovery among this population.

## 2. Material and Methods

### 2.1. Study Design and Population

The EPICA study was a multicentre, prospective, observational study of patients who have suffered a moderate to severe acute ischemic stroke. The study was approved by the local ethics committee or institutional review board of each participating centre. Inclusion criteria are ≥18 years of age, first-ever ischemic stroke within three months prior to inclusion in the study, neurologically stable at the time of inclusion, pre-stroke modified Rankin Scale (mRS) ≤1, National Institute of Health Stroke Scale (NIHSS) score between 10 and 20 at 24 hours after arrival in the ER, and diagnosis confirmed by either computerized tomography (CT) or magnetic resonance imaging (MRI). Exclusion criteria are presence of intracranial haemorrhage, other intracranial pathologies, severe systemic diseases, or cognitive deficits that may potentially interfere with the requirements of the study.

Patients were included in the study as they come for consultation or admitted to the hospital if they meet the eligibility criteria. Written informed consent was obtained from all patients included in the study. Predefined data were ascertained from patients during the course of their participation in the study at inclusion (baseline, D0), one month (M1), and three months (M3). Throughout the study period, patients received standard treatments and interventions according to the medical judgment and prescription of their respective treating physicians, including cognitive and motor rehabilitation, lifestyle recommendations, such as diet and physical activity, and use of dietary supplements.

### 2.2. Study Variables

Data were collected using a case report form, either electronically or on paper. At baseline (D0), sociodemographic and clinical history including cardiovascular risk factors were collected. Details of the index stroke gathered at the time of hospital admission were ascertained at time of inclusion, including NIHSS within 24 hours of arrival in the ER, Glasgow Coma Scale (GCS), and pre-stroke mRS. The concurrent clinical status of the patient at study inclusion was assessed and recorded, using the same assessment scales with addition of Barthel index (BI) and Mini-Examen Cognoscitivo (MEC) of Lobo [10] (a Spanish adaptation of the Mini-Mental State Examination). Results of diagnostic imaging (CT or MRI) were recorded, as well as all medications or treatment regimen (e.g., thrombolysis and drug name) received by the patient.

The following assessments were performed at M1 and M3: NIHSS, GCS, mRS, MEC, and BI. At each visit, patients were asked about their rehabilitation program frequency, lifestyle (e.g., family situation, sleeping time, and physical activity), and dietary/feeding habits including the use of dietary supplements.

### 2.3. Statistical Analysis

Sample size was calculated using tests of comparison of means between independent groups to observe significant differences of greater than 0.5 in the M3 neurological recovery between the two extreme quartiles of the sample (P25 vs. P75). Based on an alpha of 0.05 and a power of 90%, a size of 70 patients per quartile or a total sample of 280 patients was planned.

The main assessments in the study were (i) mean grade of neurological and functional recovery by comparing scores between D0, M1, and M3 overall and by subgroups according to severity based on NIHSS (i.e., NIHSS 10 − 14 = moderate, NIHSS > 14 = severe), and (ii) factors associated with higher probability of post-stroke recovery at M3.

Statistical analysis was performed using SPSS 22.0 statistical software for Windows. Baseline variables were presented using descriptive statistics. Continuous variables were described using central estimators (mean or median) and dispersion (standard deviation, SD, or interquartile range, IQR), while categorical variables were described as frequencies and percentages.

Since the planned sample size of 280 was not reached, the first to fourth quartile comparisons were substituted by median comparisons. Comparisons were made between groups using one-way analysis of variance (ANOVA) for continuous dependent variables and Pearson *χ*^2^ or Fisher's exact test for categorical dependent variables. Student *t*-test was used for variables with a normal distribution; otherwise, Wilcoxon test was used. Friedman test was used to determine the significance of evolution between visits as a whole. Results are given with *p* values and 95% confidence interval (CI).

Multivariate analyses were performed for regression analysis and ANOVA for a dependent variable by one or more variables. Factor variables divide the population in groups. The general linear model (GLM) tests the null hypotheses on the effects of other variables on the means of several groups of a single dependent variable. For regression analysis, independent variables (predictors) are specified as covariates. The procedure to generate the multivariate models was based on univariate analyses of the main variables described in the previous section to determine which variables are to be included in the multivariable analyses. Binary logistic regression models were performed for dichotomous dependent variables. Continuous and ordinal variables were transformed using dichotomous cutoffs.

## 3. Results

**Figure 1:**
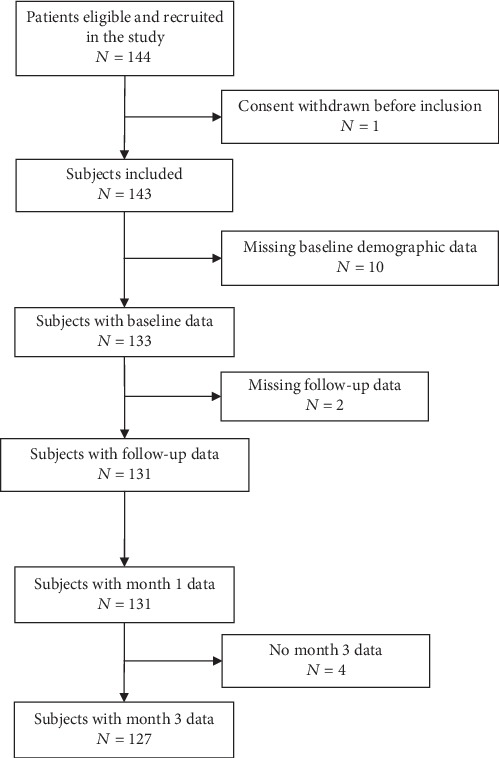
Study flow of subjects.

Twenty neurology and rehabilitation centres throughout Spain participated in this registry between April 2015 and June 2016. The study included 144 patients out of the target sample size of 280 and was terminated due to a slower than expected recruitment rate. Nonetheless, as this is an observational cohort study, the study team proceeded with the planned analyses. Thirteen patients were excluded from this analysis for various reasons, and analyses were performed on the remaining 131 patients (Figure 1). Main baseline characteristics of the study cohort are reported in Table 1. Mean age was 64.9 ± 13.8 years, with 49.2% women, and 64.3% of patients being married.

Most (87%) of patients had a pre-stroke mRS score of 0 and had moderately severe stroke (mean NIHSS score of 14.05 ± 3.8 at 24 hours after arrival at ER). Two patients had GCS of ≤9 on admission. Ischemic stroke was confirmed by CT scan in 53% and by MRI in 47% with most (40%) having partial anterior circulation infarct (PACI). The cohort risk factor profile and the different therapies received by the patients are also presented in Table 1. While centres may have different practices, rehabilitation was performed in 93% of patients, with more than three-fourths of patients receiving a regimen at least 3 times a week for at least one hour each time.

### 3.1. Neurological and Functional Recovery

The mean time between admission and inclusion into the EPICA study was 33.6 ± 30.9 days (range 0 to 154 days). The neurological and functional assessments at inclusion in EPICA (D0) are shown in Table 2. Upon inclusion in the study, median mRS score did not significantly change from admission, but NIHSS mean score had already improved by at least 3 points.

The scores in all assessment scales significantly improved from D0 to M3 (all *p* < 0.001). The overall mean NIHSS improved from 10.8 at D0 to 7.4 at M1 and 5.7 at M3. Median mRS improved from 4 at D0 to 3 at M1 and M3. Similarly, median BI improved from 40 at D0 to 59 at M1 and 70 at M3.

At D0, MEC was evaluated in only 77 (58.7%) patients, the assessment being not possible in 49 (37.4%) patients with aphasia or severe dysarthria. The mean MEC score was 29.4, corresponding to borderline to normal cognitive function compared to the possible max score of 35. At M3, there was an improvement in the ratio of correct/incorrect answers from patients as compared to D0, with an overall mean MEC score of 31.3 corresponding to a normal range.

**Figure 2:**
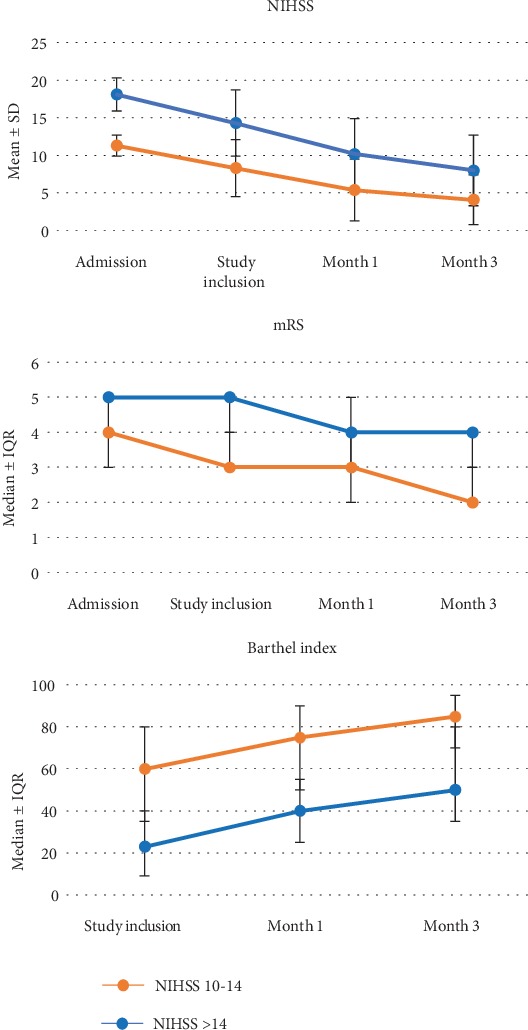
Neurological and functional recovery of stroke patients according to stroke severity subgrouped by NIHSS on admission of 10 to 14 (moderate, *n* = 77) versus 15 to 20 (severe, *n* = 54). NIHSS: National Institute of Health Stroke Scale; mRS: modified Rankin Scale; BI: Barthel index; SD = standard deviation; IQR = interquartile range.

The recovery pattern according to stroke severity, stratified according to NIHSS on admission of 10 to 14 versus >14, is shown in Figure 2. As can be seen, the improvements on neurological and functional status in the 2 groups parallel each other over the 3-month period of study observation. However, while patients with moderate severity recovered relatively well by M3, patients with more severe strokes remain with significant deficits and disabilities.

### 3.2. Predictors of Recovery

**Figure 3:**
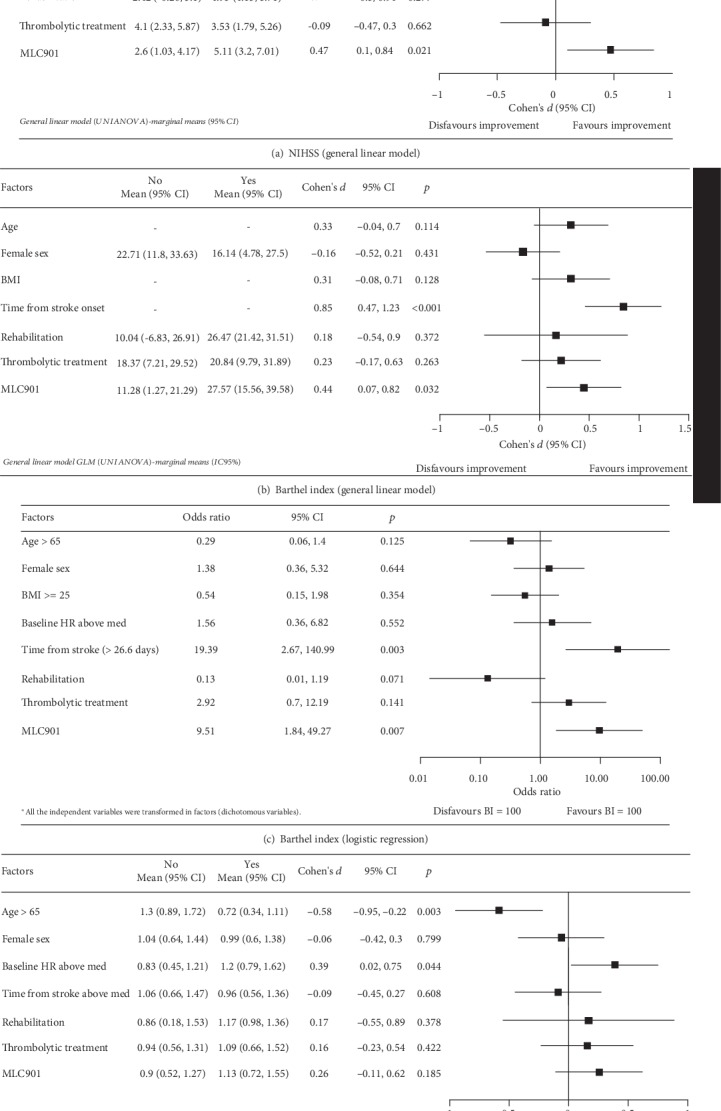
Multivariable analysis of factors influencing improvements on NIHSS (a), Barthel index (b, c), and mRS (d) at month 3.

Based on the results of an exploratory univariate analysis to select potential predictive factors of improvement (data not shown), age, sex, body mass index (BMI), time from stroke onset, baseline heart rate, treatment with thrombolysis, rehabilitation, and administration of dietary supplement MC901 were included in multivariate analysis models with M3 NIHSS, Barthel index, and mRS as dependent variables (Figure 3). Using the general linear model (GLM), time from stroke onset and use of dietary supplement MLC901 were associated with improvements in NIHSS, BI, and mRS at M3. In addition, the odds of achieving complete independence on BI on logistic regression remained in favour of MLC901 use. Patients who are older and women tend to have less improvement by M3.

On the other hand, the trends of association between recovery and the use of thrombolytic or rehabilitation were inconsistent as they may not be fully independent variables and often confounded by being prescribed based on stroke severity and other factors.

Among 40 patients who were revascularized in acute phase by thrombolysis or endovascular techniques before entering in the study, there was a nonsignificant greater percentage of NIHSS mean score improvement by D0 compared to patients who did not receive thrombolysis (33.7% vs. 19.8%; *p* = 0.09), although the trend on GLM was not in favour of thrombolytic treatment. No statistically significant differences were observed either on mRS or BI at M3, except a nonsignificant positive trend in favour of revascularization for improvement in BI (Cohen's *d* = 0.23, 95% CI -0.17–0.63) or achieving a score of 100 at M3 (OR = 2.94, 95% CI 0.71–12.23).

Similarly, rehabilitation demonstrated a nonsignificant trend towards further improvement with 12 sessions (OR = 1.63, 95% CI 0.41-6.51) and 20 sessions (OR = 1.19, 95% CI 0.34-4.17). The analysis according to the different rehabilitation intensity patterns showed no difference between groups.

**Figure 4:**
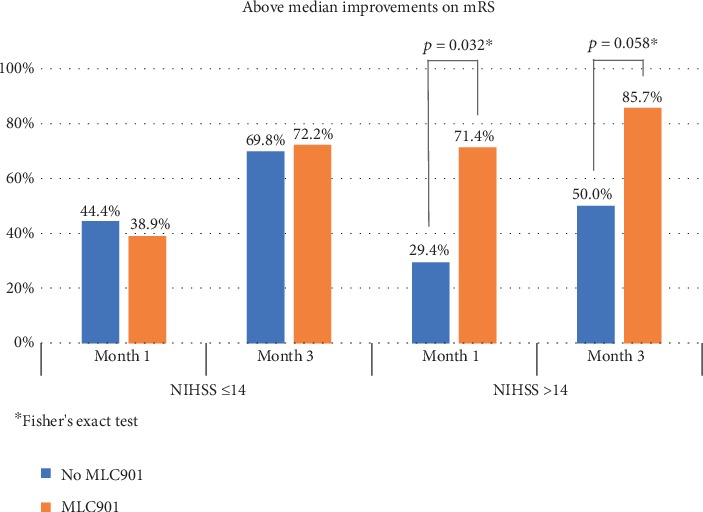
Comparison of proportions of patients who demonstrated above median improvements on modified Rankin Scale (mRS) at months 1 and 3 according to stroke severity (NIHSS score ≤14 or >14) and use of health supplement MLC901.

Given the observed effect of the use of dietary supplement MLC901 on multivariate analyses, we investigated whether a differential effect of MLC901 can be seen according to stroke severity. As shown in Table 3, baseline characteristics were well balanced between MLC901 and control groups, except a better BI median score in control arm compared to MLC901 arm (47.5 vs. 35, respectively). If the evolution at M3 showed an improvement of scores in both groups without statistical difference, more patients on MLC901 achieved an mRS above median score at M3 than in the control group, but the difference did not reach statistical significance (75.4% vs. 66.7%; *p* = 0.25). There was also a small trend in favour of MLC901 on NIHSS score (51% vs. 48%). So, we explored stroke severity as a confounding factor and then analysed the results on the stratified mRS analysis by baseline NIHSS score. The effects of MLC901 on mRS at M1 and M3 were compared after stratifying patients according to baseline NIHSS as moderate (NIHSS ≤ 14, *n* = 96) or more severe stroke (NIHSS > 14, *n* = 35). While there were no remarkable differences observed among patients with moderate stroke (NIHSS ≤ 14), a larger proportion of patients with more severe stroke (NIHSS > 14) who received MLC901 showed above median improvements on mRS at M1 (*p* = 0.032) and M3 (*p* = 0.058) compared to patients who did not (Figure 4).

## 4. Discussion

In this cohort study, we showed that patients with moderate to severe ischemic stroke overall demonstrate gradual improvement over the 3 months of follow-up as assessed on three neurological and functional scales, i.e., NIHSS, mRS, and BI. A large proportion of patients recovered from severe dependency on basic daily activities on admission and D0 to a mild or moderate disability requiring some help at M3. The improvement is more pronounced among patients with less severe stroke, i.e., NIHSS 10 to 14, than those who are more severe. While acute revascularization and rehabilitation are associated with a positive trend without achieving statistical significance, we found age and the use of dietary supplement MLC901 to be predictive of neurological and functional recovery, particularly in patients with more severe stroke.

Compared to other Spanish registries, our study population's mean age was 10 years younger than that in the Iberictus study, which reported a mean age of 75 years among the stroke patients in the Iberian Peninsula [11]. The presence of vascular risk factors in our population was similar to that observed by Moreno et al., except for a higher rate of dyslipidaemia in our study population (40.5% vs. 25.6%) [12]. This may be due to lifestyle changes in recent years, particularly poorer diet, in spite of the reported potential benefits of a Mediterranean diet in preventing cardiovascular events [13].

Based on the collected clinical information, our cohort of stroke patients appear to have received adequate diagnostic and therapeutic management. Mean delay from stroke onset to emergency room arrival was 19 hours, all patients underwent brain scanning, revascularization attempts were performed in about a third, medications prescribed were commensurate to the risk factors, and more than 90% of patients received rehabilitation.

It was satisfying to note that majority of study patients were included in a rehabilitation program soon after stroke and continued until the end of the study with more than half receiving daily sessions. While the optimal intensity is not well established, neuroimaging studies showed increased activation with increasing intensity of therapy [14]. The Canadian Stroke Guidelines recommend 3 hours per day of direct task-specific therapy, 5 days a week [15]. A Spanish study showed that improved walking speed after a multimodal rehabilitation program is associated with increased community mobility and better quality of life [16]. More recently, the interest in providing rehabilitation in group settings and potentially in a clinically “enriched environment” has increased [17, 18]. Such settings may provide the opportunity of optimizing the recovery of stroke patients, especially when the brain is “primed” by rehabilitation and combined with behaviours or interventions that promote neurorestoration.

Our study aimed to provide further data on the pattern of recovery among stroke patients particularly in those with moderate to severe deficits. Overall, our stroke patients demonstrated functional and neurological recovery over the 3-month study period. The trajectories of neurological and functional improvement appear to be similar between moderately and more severely affected patients over the 3 months of observation. While recovery from stroke is often described as complex and multifactorial, it has long been demonstrated that the initial severity of stroke is related to long-term outcomes. More recently, studies suggest that the degree of recovery is proportional to the deficits or lost function [19, 20]. Such proportion of recovery appears to be fixed at approximately 70% of the lost function [21]. Our patients with moderate severity show a pattern consistent with this hypothesis, although those with more severe strokes recovered much less. Stroke patients are known to spontaneously recover most during the first three months after a stroke, although at different rates and degrees for different baseline severity [22]. As we do not have data beyond the 3 months of observation in the study, we are unable to estimate the full extent of recovery in our patients at the time point that improvement has plateaued and beyond which no further improvement can be seen. If this “proportional recovery” hypothesis is indeed true, post-stroke recovery may be much simpler, and the main therapeutic strategy for stroke would then be to reduce stroke severity at the onset, which is the aim of revascularization and neuroprotection. The true proportionality of recovery after stroke, however, has recently been challenged [23]. Furthermore, the number of patients who would qualify for currently available acute interventions remain low, and a significant proportion of patients who received them would still have residual deficits, as was the case in our patients who received revascularization therapies. In our study, the majority of patients were included several days or weeks after stroke onset. Among these late stage cases, about one-third of them had received revascularization therapies, but their NIHSS median score at inclusion remained at 10, requiring restorative therapy. The majority of patients with stroke survives the initial event but go on to live with significant disability for many years [24]. It is, thus, important to complement acute interventional strategies that reduce brain damage with postacute phase strategies (“modifiable predictors”) that may influence long-term recovery. Restorative therapies that aim to harness neurorepair may be accessible by a large fraction of patients with stroke and, thus, hold the promise to reduce deficits and improve function for a majority of stroke survivors [7, 8, 24, 25]. As such, research on treatments to improve the quality of life of patients with chronic stroke is essential [24].

Apart from initial stroke severity, we found age and sex, both nonmodifiable factors, to be predictive of neurological and functional outcomes, similar to other reports albeit not as strongly [26–28]. Exclusion of patients with milder stroke and the smaller sample size may have reduced the impact of these predictors in our study. Likewise, we found some association between interventions, i.e., acute revascularization and postacute rehabilitation and improvement in outcomes. Both are proven, and standard therapies in stroke and the lack of significance in our study could be due to different biases. For the acute revascularization, most of our study population having been recruited at a subacute or chronic stage, patients who had benefited from an effective intervention were probably not included in the study as they did not reach the threshold of NIHSS score required at study entry. Regarding rehabilitation, because patients who did not receive it are very few (<10%), we can assume that this is a key factor in the management of global recovery we observed and remains one of the most important therapeutic recommendations in practice after a stroke, as it is in our study. Interestingly, however, we also found an association between use the health supplement MLC901 and clinical outcomes. MLC901 (NurAiD™II) is a dietary supplement marketed in Europe since 2011 and introduced in Spain in 2014. Animal and cell studies have shown that MLC901 stimulated natural processes of neural repair and neuroplasticity [29, 30]. Clinical studies showed that its precursor, MLC601, improved motor, visual, and functional outcomes in patients with stroke at 3 months and up to 2 years [31–33]. Subanalyses of the large clinical trial showed larger treatment effects among patients with predictors of poor outcome, including worse baseline NIHSS, likely because they had more potential to benefit from treatment [26, 34, 35]. A more recent analysis demonstrated that among patients who perform persistent rehabilitation, the use of this dietary supplement increased the rate of functional independence at 3 months and sustained over 2 years compared to rehabilitation alone [36]. This benefit was not seen among patients who did not consistently perform rehabilitation, suggesting that neurorestorative therapies work better when the brain is primed by rehabilitation. Our analyses showing the use of MLC901 being associated with improved outcomes at 3 months on neurological and functional scales are consistent with these studies. Furthermore, we found more remarkable improvement on mRS as early as one month with MLC901 among patients with more severe strokes than in patients who are less severe.

Our study certainly has several limitations. We did not achieve the originally planned sample size due to poorer than expected recruitment. Excluding mild strokes in our cohort study may have affected recruitment. As described, we adjusted the statistical approach to be more appropriate for our eventual sample size. Yet, the decreased power in the study likely reduced our ability to detect statistical significance. Nonetheless, we observed trends in the same direction on several endpoints, which may support clinical effects that deserve further research. In addition, the study was designed with a 3-month follow-up to enable us to observe the speed of recovery of stroke patient during this period, but it did not allow us to see the full extent, durability, and rate of delayed recovery or deterioration [37]. A large Swedish registry showed that transition from independence in activities of daily living to dependency between 3 and 12 months after a stroke occurs in a high proportion of patients between 3 and 12 months [38]. Nevertheless, our study contributes further data on the pattern and predictors of neurological and functional recovery in patients with moderate to severe stroke over 3 months, particularly in the Spanish population.

## 5. Conclusions

In conclusion, our study in a cohort of patients with moderate to severe stroke shows overall recovery on neurological and functional assessments during the 3 months of study observation. We demonstrated similar trajectories, but not degree, of recovery between patients with moderate and those with more severe stroke. These findings may have implications on designing studies aimed at neurorestoration, recovery, and rehabilitation. Apart from demonstrating traditional “non-modifiable” predictors of outcome after stroke, like age, sex, and stroke severity, we also detected association between the use of health supplement MLC901 and recovery that is consistent with published reports and worth exploring in future studies.

## Figures and Tables

**Table 1:** Baseline characteristics of study population.

Characteristics	*N* = 131
Demographics	
Age (years)	64.9 ± 13.8
Women	64 (49.2)
Married	83 (64.3)
Weight (kg)^∗^	75.2 ± 13.2
Height (cm)^∗^	166.7 ± 9.9
Body mass index (kg/m^2^)^∗^	27.1 ± 4.4
Risk factors	
Hypertension	77 (58.8)
Hyperlipidemia	53 (40.5)
Diabetes mellitus	30 (22.9)
Atrial fibrillation	16 (12.2)
Ischemic heart disease	12 (9.2)
Smoking	36 (27.5)
Alcohol consumption	10 (7.6)
Drug abuse	4 (3.1)
Prestudy clinical information	
Pre-stroke modified Rankin Scale	
0	113 (86.9)
1	17 (13.1)
Stroke onset to arrival in emergency room (hours)^∗∗^	18.9 (28.6)
National Institute of health stroke scale at 24 hours from admission^∗∗∗^	14.1 (3.8)
Modified Rankin Scale on admission, (median, IQR)^∗∗∗∗^	4 (3-5)
Glasgow coma scale on admission^∗∗^	14.1 (1.9)
Brain imaging performed	
Computed tomography	70 (53.4)
Magnetic resonance imaging	61 (46.6)
Type of lesion^∗∗∗∗^	
Partial anterior circulation infarct (PACI)	51 (39.5)
Total anterior circulation infarct (TACI)	38 (29.5)
Lacunar infarct (LACI)	23 (17.8)
Posterior circulation infarct (POCI)	17 (13.2)
Stroke onset to study entry; days	33.6 ± 30.9
Early hyperacute stage (0 to 6 hours)	6 (4.6)
Late hyperacute stage (<6 to24hours)	5 (3.8)
Acute stage (>24 h to 72 hours)	12 (9.2)
Post-acute stage (>3 to 7 days)	15 (11.5)
Subacute stage (1 to 3 weeks)	21 (16.0)
Chronic stage (>3 weeks)	72 (55.0)
Therapies received	
Revascularization (intravenous thrombolysis, endovascular thrombectomy)	40 (30.5)
Medications by class^∗^	
Statin & other lipid-lowering agents	65 (51.6)
Antihypertensive	60 (47.6)
Betablocker^∗^	20 (15.9)
Antidiabetic	20 (15.9)
Antiplatelet	55 (43.7)
Anticoagulant	40 (31.7)
Antiulcer	41 (32.5)
Antidepressant	22 (17.5)
Anxiolytic/sleeping pill	22 (17.5)
Analgesic	8 (6.3)
Antiarrhythmic	5 (4.0)
Antiepileptic	5 (4.0)
Rehabilitation^∗∗∗^	119 (93.0)
≥1 hour daily	67 (56.8)
≥1 hour, 3 times a week, <1 hour daily	26 (22.0)
Other	25 (21.2)
Dietary supplementation	60 (45.8)
MLC901 (NurAiD™)	59 (45.0)
Citicoline (Somazina®)	2 (1.5)

Data missing in ^∗^5 patients, ^∗∗^3 patients, ^∗∗∗^1 patient, ^∗∗∗∗^2 patients. Unless otherwise stated, summary data are presented as *n* (%) or as mean ± standard deviation.

**Table 2:** Neurological and Functional Assessments at Baseline, month 1 and month 3.

Assessments	Baseline (D0)	Month 1	Month 3	*p* value^∗^
NIHSS	*n* = 131	*n* = 131	*n* = 126	
Mean (SD)	10.8 (5.0)	7.4 (4.9)	5.7 (4.3)	*p* < 0.001^1^
Median (IQR)	10 (7 - 15)	7 (4 -10)	5 (2 - 9)	*p* < 0.001^2^
Score > 14; *n* (%)	35 (26.7)	15 (11.5)	6 (4.8)	
mRS	*n* = 131	*n* = 131	*n* = 127	
Median (IQR)	4 (3-5)	3 (3-4)	3 (2-4)	*p* < 0.001^3^
0; *n* (%)	2 (1.5)	2 (1.5)	4 (3.1)	*p* < 0.00001^4^
1	3 (2.3)	4 (3.1)	11 (8.4)	
2	8 (6.1)	22 (16.8)	41 (31.3)	
3	30 (22.9)	41 (31.3)	35 (26.7)	
4	45 (34.4)	40 (30.5)	25 (19.1)	
5	43 (32.8)	22 (16.8)	10 (7.6)	
6	0 (0.0)	0 (0.0)	1 (0.8)	
BI	*n* = 127	*n* = 126	*n* = 122	
Mean (SD)	45.1 (29.2)	58.9 (28.17)	69.9 (27.7)	*p* < 0.001^1^
Median (IQR)	40 (20-70)	55 (40-85)	80 (50-95)	
95-100	8 (6.3)	17 (13.4)	31 (25.4)	
65-90	31 (24.4)	44 (34.6)	50 (41.0)	
≤60	88 (69.3)	65 (51.2)	41 (33.6)	
GCS	*n* = 131	*n* = 130	*n* = 127	
Mean (SD)	14.4 (1.29)	14.6 (1.02)	14.8 (1.18)	*p* < 0.001^1^
MEC	*n* = 77	*n* = 74	*n* = 82	
Mean (SD)	29. 4 (7.1)	31.4 (5. 7)	31.3 (6. 2)	*p* < 0.001^1^
Median (IQR)	32 (28-35)	34 (30-35)	35 (30-35)	

^∗^D0 versus month 3. ^1^ Student t-test for repeated measures. ^2^ McNemar test. ^3^ Wilcoxon test. ^4^ Mann-Whitney U test for ordinal analysis. NIHSS: National Institute of Health Stroke Scale; mRS: modified Rankin Scale; BI: Barthel index; GCS: Glasgow Coma Scale; MEC: Mini-Examen Cognoscitivo de Lobo; SD: standard deviation; IQR: interquartile range.

**Table 3:** Comparison of MLC901 vs. control on prognostic factors, Neurological and Functional Assessments at Baseline.

Baseline characteristics, *n*	MLC901 *n* = 59	Control *n* = 72
Age, years, mean (SD)	62.8 (13.9)	66.7 (14.0)
Women, *n* (%)	28 (47.5)	36 (50.0)
Body mass index (kg/m^2^), mean (SD)	27.4 (4.7)	26.8 (4.2)
Stroke onset to emergency room arrival, hours, mean (SD)	20.6 (31.0)	18.4 (26.7)
NIHSS on admission, median (IQR)	12 (10, 18)	12 (11, 17)
GCS on admission, median (IQR)	15 (13, 15)	15 (14, 15)
Thrombolysis at admission, *n* (%)	19 (32.2)	21 (29.2)
NIHSS at baseline, median (IQR)	10 (8, 16)	10 (7, 14)
mRS at baseline, median (IQR)	4 (3, 5)	4 (3, 5)
BI at baseline, median (IQR)	35 (20, 65)	47.5 (25, 70)

NIHSS: National Institute of Health Stroke Scale; GCS: Glasgow Coma Scale; mRS: modified Rankin Scale; BI: Barthel index; SD: standard deviation; IQR: interquartile range.

## Data Availability

All the data used to support the findings of this study are included within the article.

## Abbreviation

EPICA: Estudio sobre la recuperación funcional de los PacIentes con Accidente vascular cerebral isquémico Agudo de gravedad intermedia.
